# A multi-institutional CT practices survey of pediatric head, chest, and abdomen-pelvis examinations

**DOI:** 10.1177/20584601251340974

**Published:** 2025-05-12

**Authors:** Elena Tonkopi, Megan Iwaskow, Cecilie Karlstad Lønningen, Alex Myrvold Johansen, Sivethan Suganthan, Yulia Kotlyarova, Mohamed Badawy, Catherine Gunn, Jessica Kimber, Dana Jackson, Mercy Afadzi Tetteh, Tanja Oestgaard Holter, Safora Johansen

**Affiliations:** 1 Department of Diagnostic Radiology, 3688 Dalhousie University, Halifax, Canada; 2 Department of Radiation Oncology, 3688 Dalhousie University, Halifax, Canada; 3 Department of Diagnostic Imaging, 432234 Nova Scotia Health, Halifax, Canada; 4 School of Health Sciences, 3688 Dalhousie University, Halifax, Canada; 5 Department of Diagnostic Imaging, 3682 IWK Health, Halifax, Canada; 6 Health Faculty, 60499 Oslo Metropolitan University, Oslo, Norway; 7 Department of Economics, 3688 Dalhousie University, Halifax, Canada; 8 569173 Monash Health Imaging, Monash Health, Clayton, Australia; 9 Department of Medical Imaging and Radiation Sciences, School of Primary and Allied Health Care, Faculty of Medicine, Nursing and Health Sciences, 2541 Monash University, Clayton, Australia; 10 Department of Diagnostic Imaging, 60483 Akershus University Hospital, Loerenskog, Norway; 11 Department of Physics and Computational Radiology, 155272 Oslo University Hospital, Oslo, Norway; 12 Department of Cancer Treatment, 155272 Oslo University Hospital, Oslo, Norway; 13 Health and Social Science Cluster, Singapore Institute of Technology, Singapore

**Keywords:** pediatric CT, diagnostic reference levels, radiation dose

## Abstract

**Background:** Pediatric patients are particularly vulnerable to the stochastic effects of ionizing radiation. Despite these risks, CT remains diagnostically essential in pediatric care. Diagnostic reference levels (DRLs) have been recommended as a radiation dose optimization tool to address these concerns. **Purpose:** This study aims to survey pediatric CT practices at different facilities in Australia, Canada, and Norway and to suggest local DRLs (LDRLs) at each facility as a baseline for future surveys. **Materials and methods:** Radiation dose indices, imaging, and demographic data were collected retrospectively at each facility using PACS for unenhanced CT head, contrast-enhanced chest, and contrast-enhanced abdomen-pelvis examinations in patients from 0 to 15 years of age. The LDRL values were determined for CT dose indices and size-specific dose estimate (SSDE) values. The Kruskal–Wallis test assessed the equality of populations across countries for all dosimetric quantities. Ordinary least squares regression was employed to express SSDE as a linear function of patient weight. **Results:** The LDRLs for Australian, Canadian, and Norwegian facilities were determined and examined for each age group. Canadian and Norwegian LDRL data were most similar, with Australian values being comparatively lower for all categories except for 11–15-year-old abdomen-pelvis examinations. The SSDE and patient weight were significantly positively correlated for each examination/country combination. **Conclusion:** The proposed local reference levels can provide local baselines for dose optimization and continuous dose assessment.

## Introduction

Advancements in image quality, relatively short scan times, and increased availability of computed tomography (CT) have led to increased utilization of the imaging modality globally.^1^ However, CT examinations contribute more to a patient’s ionizing radiation exposure than other radiographic imaging modalities.^2^ Pediatric patients are particularly vulnerable to the adverse effects of ionizing radiation exposure, such as the increased lifetime risk of cancer, due to their longer life expectancy and rapid cell replication and development.^1–5^ Ultimately, CT remains an essential diagnostic tool in pediatric medicine, highlighting the need to monitor examination doses and minimize radiation exposure in this population.

Diagnostic reference levels (DRLs) introduced by the International Commission on Radiation Protection (ICRP) in 1996 are an optimization tool to ensure that the use of ionizing radiation is As Low As Reasonably Achievable (ALARA principle) without sacrificing diagnostic image quality.^2,6^ The ICRP defines a DRL value as the 75^th^ percentile of the distribution of median doses collected from several facilities.^2^ Local DRLs (LDRLs) are defined by the European Commission as the 75^th^ percentile of pooled dose distribution in a single large facility or 2–3 small facilities.^6^ LDRLs can be compared to available national or international DRLs to optimize dose, achieving exam exposures that adhere to the ALARA principle while maintaining the image quality required for diagnostic interpretation. The European Commission strongly recommends updating NDRLs and LDRLs regularly at least every 3–5 years, and comparison of patient dose levels of a hospital or a group of hospitals should be carried out at the minimum frequency of once per year.^6^

Whereas adult DRLs are based on the average adult’s expected radiation dose under a given imaging protocol, assigning DRLs to pediatric CT examinations poses the extra challenge of identifying the ‘average’ patient. Pediatric DRLs, therefore, require the subdivision of the population into smaller representative cohorts.^6^ Pediatric DRLs have historically been grouped into age cohorts, the most common cohorts being <1, 1–5, 6–10, and 11–15 years.^2^

The volume computed tomography dose index (CTDI_vol_) and dose length product (DLP) represent radiation dose metrics traditionally used to express DRLs in CT.^2,6–11^ Other variables, such as size-specific dose estimate (SSDE), can be incorporated into DRLs, especially when discussing their utilization in pediatric imaging.^2,6^ Whereas CTDI_vol_ does not consider patient size, SSDEs seek to combine the actual size of the patient and the measured CTDI_vol_ to produce a figure that more accurately describes the patient’s dose.^12^ SSDE is recommended as a dose indicator for pediatric patients, but there are limitations to its use related to dose current modulation and variable patient thickness.^6^

This collaborative study of three different institutions from Australia, Canada, and Norway aims to survey pediatric CT practices at local health facilities. These countries represent diverse healthcare systems and demographic profiles, which can offer valuable insights into the variability and optimization potential of radiation doses in pediatric CT imaging. Although our surveys do not represent nationwide data from each country, they allow us to determine local reference levels for included facilities. The academic relationship between institutions facilitated access to data, enabling a comprehensive multi-institutional analysis that might not have been feasible otherwise. By comparing practices sampled from these countries, we aim to identify commonalities and differences that can inform global standards and contribute to the ongoing efforts in radiation safety in pediatric imaging.

## Materials and methods

### Data collection

CT scan data from pediatric examinations in patients ranging from 0 to 15 years of age were collected retrospectively from each facility using Picture Archiving and Communication Systems (PACS). Regional Sectra IDS7 PROD (Linköping, Sweden) was used in Norway, Agfa Enterprise Imaging (Agfa HealthCare, Mortsel, Belgium) in Canada, and Philips Vue PACS (Philips Healthcare, Best, Netherlands) in Australia. In Norway, data were collected from the largest public hospital responsible for the majority of pediatric diagnostic examinations in the country. In Canada, data were collected from a public dedicated pediatric hospital providing primary, secondary, and tertiary care to children across three Canadian provinces. In Australia, data were collected from a public primary care facility network, including a large pediatric hospital and other satellite sites. Pediatric CT at the surveyed Australian facilities was used only as ancillary imaging following MRI or ultrasound examinations. The surveyed examinations included the unenhanced head, contrast-enhanced chest, and contrast-enhanced abdomen-pelvis CTs. Patients were organized into groups by age and exam type; age groupings were <1 year, 1–5 years, 6–10 years, and 11–15 years. Data collected from each scan included patient age and thickness, CTDI_vol_, DLP, and patient weight for chest and abdomen-pelvis scans. The CTDI_vol_ refers to a 16 cm phantom for the head examination and a 32 cm phantom for body scans. The anterior to posterior (AP) thickness was measured at a level immediately superior to the glabella in head examinations, the level of the carina in chest examinations, and the level of the mid-kidney in abdominopelvic examinations. The study aimed to gather data for at least 30 patients in each age category and exam type. Due to the conservative use of CT in pediatrics, achieving this sample size required CT examinations from multiple years to be considered. The surveyed examination dates were limited to the period of 2016–2024 to ensure the data were relatively recent, representative of the current imaging protocols, and available on the associated data storage systems. Table 1 lists the CT scanners used in this study.

**Table 1.** Characteristics of CT scanners included in the survey.

Hospital	Manufacturer	Model	Install year	Detector configuration^a^	Iterative reconstruction
Australian	GE	Discovery 750HD	2007	64 × 0.625	ASiR-V, VEO
	Philips	iCT	2015	128 × 0.625	iDose, IMR
	Canon	Aquilion One	2008	320 × 0.5	AiDR 3D
	Siemens	Somatom Force^b^	2016	2 × 96 × 0.6	ADMIRE
Canadian	Canon	Aquilion Prime	2014	80 × 0.5	AiDR
Norwegian	Siemens	Somatom Force^b^	2019	2 × 96 × 0.6	ADMIRE

^a^Detector rows x detector element thickness.

^b^Dual source.

### Data analysis

Descriptive statistics were computed using Microsoft Excel (Microsoft Corporation, Redmond, WA, USA). Microsoft Excel was used to plot SSDE values versus patient weight for chest and abdomen/pelvis examinations and to estimate and plot the fitted values of SSDE as linear functions of weight using ordinary least squares. SSDE was calculated using conversion Tables 1(C) and 2(C) from the American Association of Physicists in Medicine (AAPM) Report 204.^12^ As recommended by the European Commission, LDRL values were presented as the 75^th^ percentile of CTDI_vol_, DLP, and SSDE distributions.^6^ Comparisons were made to previously established National DRLs from Canada, Australia, and Nordic countries.^13–15^

**Table 2.** Median values with interquartile ranges in the brackets for weight, age, AP thickness, and radiation dose metrics (CTDI_vol_, DLP, SSDE) for different patient age groups.

Hospital	Exam	Age	*N* ^a^	Weight, kg	Thickness, cm	CTDI_vol_, mGy	DLP, mGy·cm	SSDE, mGy
Australian	Head	<1	30	–	15.6 (14.1–16.3)	9.1 (7.0–9.6)	136.7 (106.9–155.6)	–
		1–5	30	–	17.8 (16.9–18.2)	10.8 (10.2–12.1)	177.7 (163.2–206.0)	–
		6–10	30	–	18.2 (17.6–18.7	14.5 (13.2–15.8)	255.9 (238.9–281.9)	–
		11–15	30	–	19.0 (18.7–19.6)	20.9 (18.6–22.7)	365.1 (321.1–398.5)	–
	Chest	<1	30	5.7 (3.8–8.1)	9.9 (9.2–11.0)	0.4 (0.4–0.6)	8.1 (6.6–9.9)	1.0 (0.9–1.5)
		1–5	30	17.0 (15.0–19.0)	14.2 (13.3–15.1)	0.5 (0.5–0.6)	12.7 (11.8–15.8)	1.1 (1.0–1.2)
		6–10	30	25.2 (21.8–32.8)	16.8 (16.1–18.2)	0.8 (0.7–0.9)	20.8 (17.3–25.6)	1.4 (1.2–1.6)
		11–15	30	45.9 (39.6–53.4)	20.4 (18.6–22.3)	1.1 (1.0–1.3)	34.2 (30.5–42.7)	1.7 (1.5–1.9)
	Abd/pelvis	<1	7	6.0 (5.7–6.9)	11.3 (9.8–12.3)	0.7 (0.6–0.7)	13.9 (11.1–15.5)	1.4 (1.2–1.8)
		1–5	30	15.6 (13.9–17.9)	15.1 (14.3–16.3)	0.8 (0.7–1.0)	27.1 (19.2–39.4)	1.6 (1.3–1.9)
		6–10	30	29.9 (22.1–34.8)	17.6 (16.6–19.5)	1.3 (1.1–1.7)	54.2 (47.8–70.6)	2.3 (2.1–2.8)
		11–15	30	50.0 (43.3–59.5)	21.4 (19.8–22.8)	2.8 (2.1–4.6)	131.3 (94.2–230.1)	4.0 (3.2–6.0)
Canadian	Head	<1	30	–	13.5 (12.6–15.8)	30.9 (30.9–30.9)	496.8 (468.1–539.3)	–
		1–5	30	–	17.4 (16.9–17.7)	31.4 (30.9–31.4)	575.8 (536.6–610.7)	–
		6–10	30	–	18.3 (17.7–19.2)	34.9 (34.9–34.9)	699.3 (682.9–717.8)	–
		11–15	30	–	19.2 (18.6–19.8)	41.8 (34.9–41.8)	795.2 (722.2–858.6)	–
	Chest	<1	30	6.4 (5.1–7.6)	9.9 (9.3–10.2)	1.5 (1.3–1.7)	27.0 (23.3–29.8)	3.6 (3.2–4.1)
		1–5	30	13.6 (12.7–15.4)	11.6 (10.7–11.9)	1.8 (1.6–1.8)	39.0 (35.5–44.8)	3.9 (3.6–4.3)
		6–10	30	25.8 (23.6–28.5)	14.0 (13.3–14.8)	2.4 (2.1–2.7)	67.1 (58.4–75.6)	4.8 (4.2–5.2)
		11–15	30	39.3 (36.6–47.3)	16.2 (15.7–17.2)	2.8 (2.4–3.0)	92.1 (79.6–101.9)	4.9 (4.3–5.5)
	Abd/Pelvis	<1	11	5.5 (4.1–6.4)	10.1 (9.4–10.9)	1.8 (1.6–1.9)	48.4 (35.0–51.6)	4.3 (3.9–4.6)
		1–5	30	14.4 (12.0–16.0)	13.6 (12.8–14.5)	2.1 (1.8–2.4)	70.3 (59.7–76.4)	4.1 (3.7–4.9)
		6–10	30	23.8 (22.3–26.4)	15.8 (14.4–16.6)	2.4 (2.2–2.8)	98.2 (85.0–107.4)	4.4 (4.2–5.1)
		11–15	30	41.0 (36.6–48.5)	16.8 (15.9–19.1)	3.0 (2.5–3.6)	130.7 (105.7–169.2)	4.9 (4.5–5.7)
Norwegian	Head	<1	23	–	12.8 (11.7–13.6)	29.1 (4.9–30.0)	430.4 (74.4–480.7)	–
		1–5	28	–	16.2 (15.1–16.6)	34.1 (33.4–36.8)	610.4 (557.3–658.2)	–
		6–10	30	–	17.5 (16.8–17.9)	36.6 (32.9–40.9)	674.6 (563.6–791.2)	–
		11–15	30	–	17.9 (17.4–18.2)	40.9 (40.3–41.3)	759.3 (717.2–809.7)	–
	Chest	<1	30	4.1 (3.0–7.0)	9.4 (8.5–10.5)	0.8 (0.6–1.1)	12.2 (9.7–18.9)	1.9 (1.4–2.5)
		1–5	30	15.0 (12.3–15.4)	11.2 (10.6–11.7)	1.1 (0.9–1.6)	22.2 (19.1–34.7)	2.6 (2.2–3.4)
		6–10	27	26.6 (24.0–31.0)	12.9 (12.2–14.3)	1.7 (1.4–2.1)	44.8 (36.6–55.1)	3.4 (2.8–4.2)
		11–15	30	47.0 (42.0–55.0)	15.7 (14.5–17.5)	2.0 (1.7–2.9)	61.2 (53.0–97.9)	3.7 (3.2–5.6)
	Abd/Pelvis	<1	23	6.0 (4.1–8.0)	12.9 (10.6–13.5)	0.9 (0.5–1.1)	20.5 (9.6–29.6)	1.8 (1.3–2.2)
		1–5	29	11.0 (8.0–13.0)	13.8 (13.0–15.1)	1.2 (1.1–1.5)	41.4 (27.2–54.5)	2.4 (2.1–3.0)
		6–10	22	25.5 (20.3–28.0)	16.9 (15.5–18.1)	2.4 (1.6–3.7)	88.0 (63.1–119.2)	4.2 (2.8–5.7)
		11–15	22	44.7 (38.4–57.0)	19.5 (18.5–21.1)	5.1 (3.3–8.1)	225.8 (151.8–372.9)	8.0 (5.3–10.8)

^a^*N* is the number of patients.

STATA/SE, Version 18.0 (StataCorp, College Station, TX, USA) was used to perform the Kruskal–Wallis test and post-hoc Dunn’s test^16^ with Bonferroni adjustment, to assess the equality of populations for all measures of radiation exposure. STATA was also used to calculate the Pearson correlation coefficient (R) and corresponding *p*-value for SSDE and weight in chest and abdomen-pelvis CT scans.

## Results

**Figure 1.**
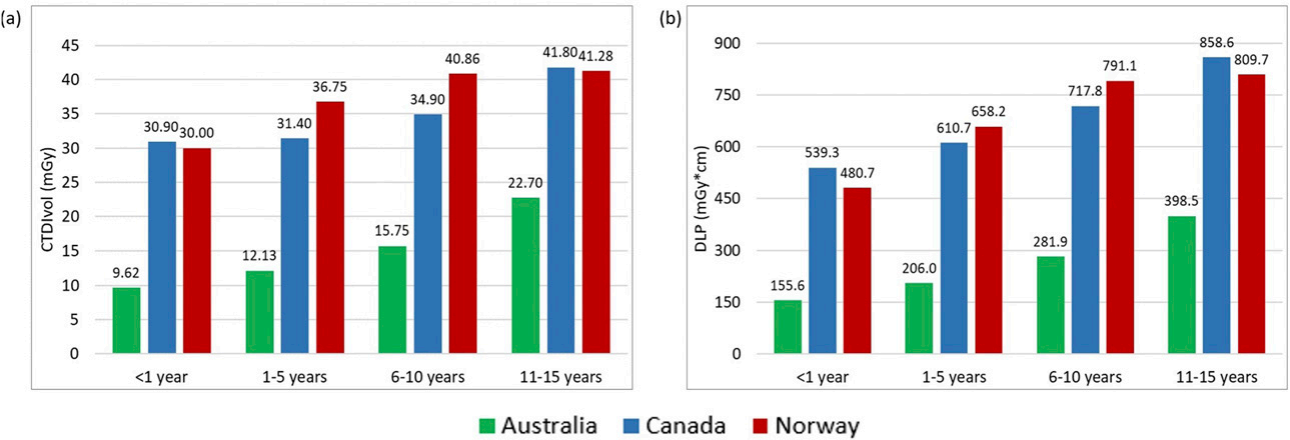
The 75^th^ percentile of the CTDIvol (a) and DLP (b) values for Head CT examinations in different age groups.

**Figure 2.**
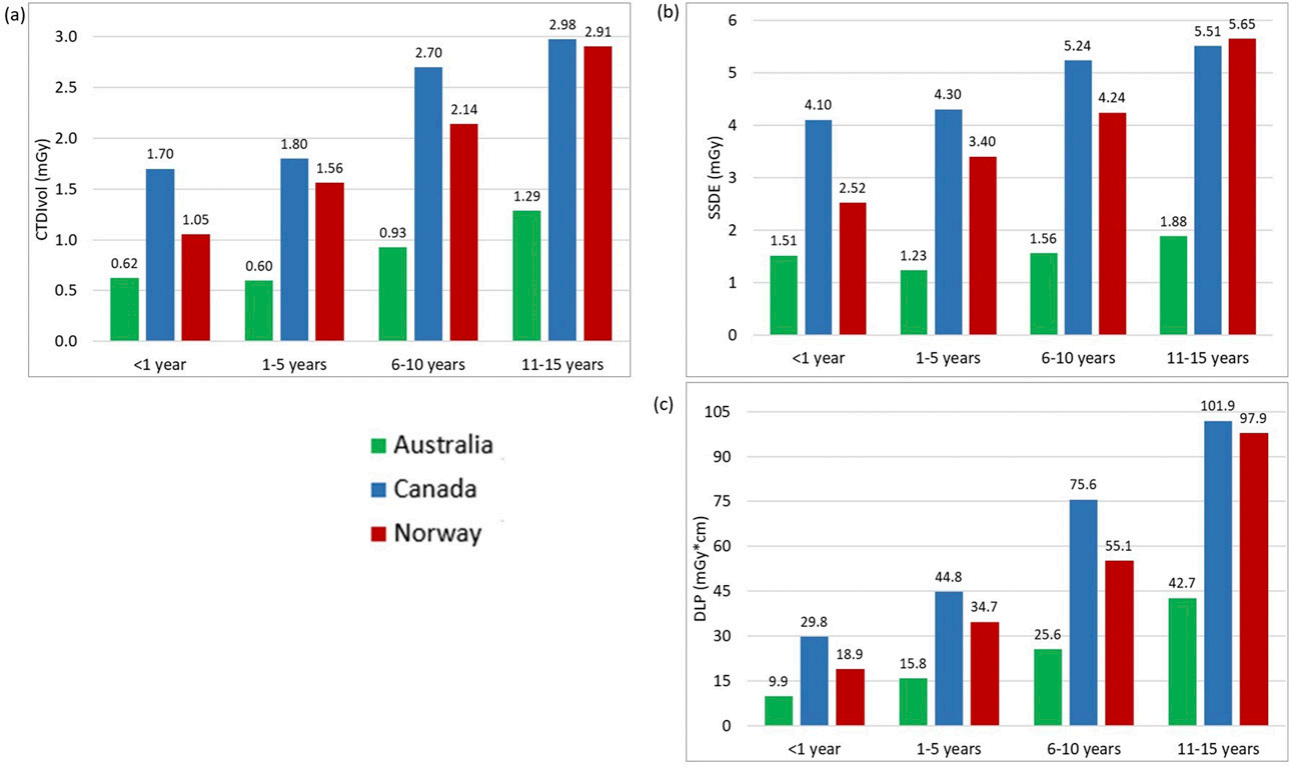
The 75^th^ percentile of the CTDIvol (a), SSDE (b), and DLP (c) values for chest CT examinations in different age groups.

**Figure 3.**
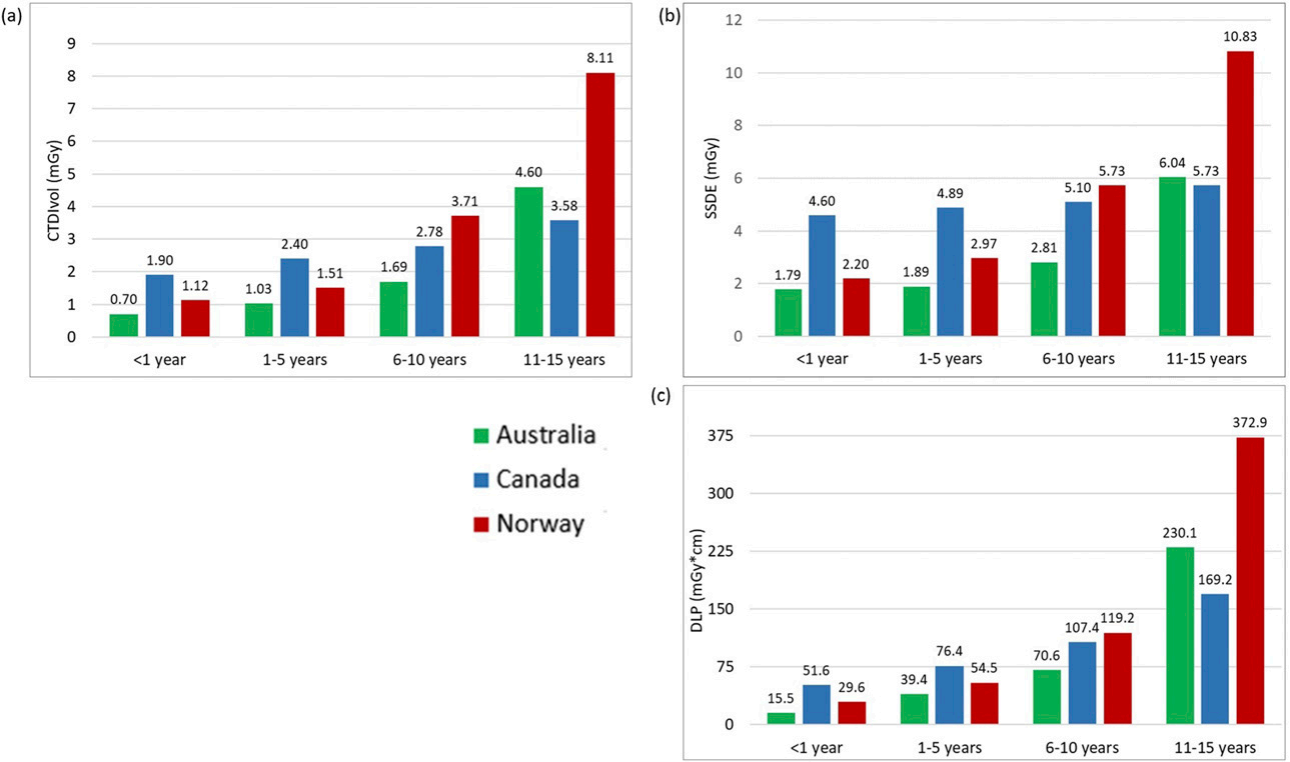
The 75^th^ percentile of CTDIvol (a), SSDE (b), and DLP (c) values for abdomen-pelvis CT examinations in different age groups.

Table 2 displays the descriptive statistics for all examinations and age groups, including median and interquartile ranges for the CTDI_vol_, DLP, and SSDE values. The 75^th^ percentile graphs are shown in Figures 1–3, representing the proposed local DRL values for each facility. LDRLs increased with the increasing age, except for Australia’s results for chest CT in <1 year-olds, where SSDE and CTDI_vol_ values were, respectively, 23% and 4% higher in <1-year patients than in the 1–5-year-old group (Fig. 2). The LDRL values from Australian facilities were the lowest in all comparisons except for the 11–15-year-old abdomen-pelvis scans, where the Canadian hospital had the lowest dose values (Figure 3). The CTDI_vol_ and DLP values from the Canadian hospital exceeded those from Norwegian and Australian facilities for chest CT in every age range (Figure 2). For abdominopelvic examination, the Canadian hospital demonstrated the highest values in the two youngest patient groups; however, dose indices for 6–15-year-old patients were the highest at the Norwegian hospital (Figure 3).

The Kruskal–Wallis test found significant differences in CTDI_vol_, DLP, and SSDE distributions for all examinations and age groups across countries (all *p*-values <0.002). The comparison between Canadian and Norwegian hospitals using post-hoc Dunn’s test with Bonferroni adjustment showed a significant difference at the 5% significance level for most age groups and examinations. No significant differences were demonstrated between Canadian and Norwegian facilities in all dosimetric indices for abdomen-pelvis CT in the 6–10-year-old age range with *p*-values of 0.601, 0.359, and 0.314 for CTDI_vol_, DLP, and SSDE, respectively. For head examinations, there were no significant differences in DLPs for patients from 1 to 15 years (*p* = .298 for 1–5, *p* = .643 for 6–10, and 0.429 for 11–15 years old), as well as in the CTDI_vol_ values for patients of 6 to 15 years (*p* = 1.000 for 6–10 and *p* = .615 for 11–15 years old). The only non-significant difference with *p*-value >.05 for chest scans was found for all three dosimetric indices in the 11–15-year group (*p*-values were 0.147, 0.071, and 0.331 for CTDI_vol_, DLP, and SSDE, respectively).

The comparison based on post-hoc Dunn’s test with Bonferroni adjustment for Australian and Norwegian hospitals showed a significant (at the 5% level) difference for all dosimetric quantities across all exams and age groups except for the abdominopelvic DLPs for 1–5 years (*p* = .076) and all dosimetric indices for the abdomen/pelvis in the youngest group, which contained only seven observations from Australia, with *p*-values of 0.434, 0.227, and 0.638 for CTDI_vol_, DLP, and SSDE, respectively. Performing the test between Australian and Canadian hospitals revealed a significant difference between most examinations and age groups, except for the abdominopelvic CT in the 11–15 years category: CTDI_vol_ (*p* = .921), DLP (*p* = 1.000), and SSDE (*p* = .078).

**Figure 4.**
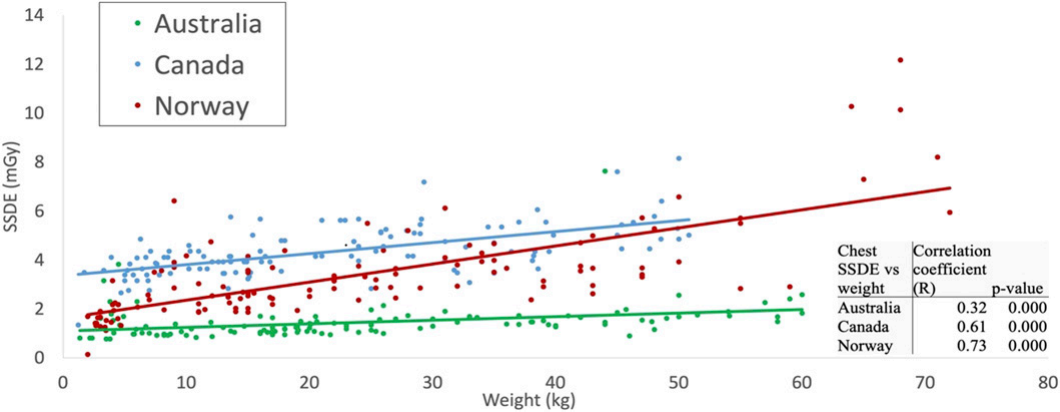
Scatter-plot comparison and correlation coefficients for pediatric chest CT SSDE and patient weight for surveyed hospitals in each country. The resulting fitted functions are as follows: SSDE_Australia_ = 1.098 + 0.014·weight; SSDE_Canada_ = 3.357 + 0.045·weight; SSDE_Norway_ = 1.617 + 0.074·weight.

**Figure 5.**
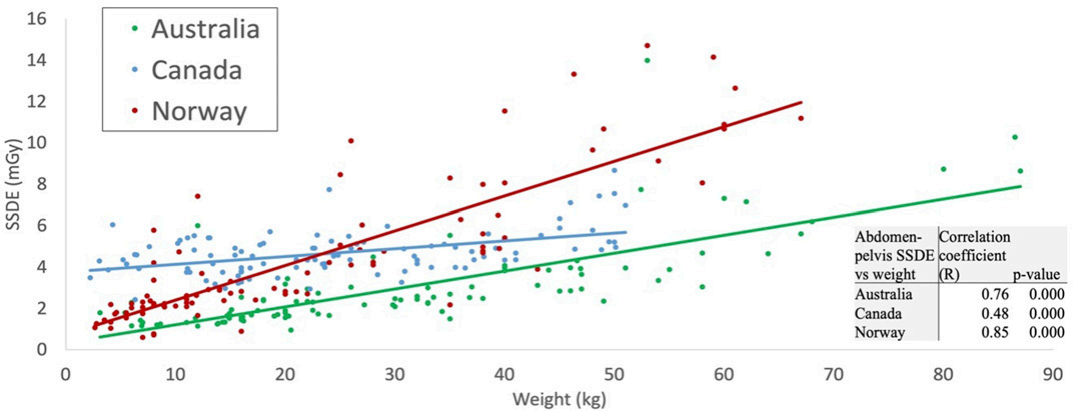
Scatter-plot comparison correlation coefficients for pediatric abdomen-pelvis CT SSDE and patient weight for surveyed hospitals in each country. The resulting fitted functions are as follows: SSDE_Australia_ = 0.342 + 0.087·weight; SSDE_Canada_ = 3.756 + 0.038·weight; SSDE_Norway_ = 0.724 + 0.168·weight.

Using ordinary least squares, the fitted values of SSDE in the chest and abdomen-pelvis CT examinations were estimated as linear functions of patient weight (Figures 4 and 5). For each examination/country combination, the SSDE and patient weight were significantly positively correlated. The Pearson correlation coefficients for pediatric chest CT were 0.61, 0.73, and 0.32 for Canadian, Norwegian, and Australian hospitals, respectively (Figure 4). The correlation coefficients for pediatric abdomen-pelvis CT were 0.48 for Canada, 0.85 for Norway, and 0.76 for Australia (Figure 5). All correlation coefficients were statistically different from zero (*p*-values <0.05).

## Discussion

This study investigated pediatric CT practices and suggested local diagnostic reference levels at different facilities providing pediatric care in three countries. None of those facilities previously conducted this type of survey; therefore, our results can provide a baseline for future studies. It should be noted that the surveyed Australian facilities used CT as ancillary imaging to monitor, in most cases, oncologic disease diagnosed using a different modality. In brain imaging, CT was employed only for craniosynostosis, or trauma to diagnose a bleed or fractures. Depending on the results, those patients would be followed with an MR scan for further evaluation. Therefore, a higher noise level was acceptable for CT studies at the Australian facility, unlike at the Canadian and Norwegian hospitals included in this survey.

The LDRL values determined from Australian data were below the previously established NDRLs.^13^ While Australian NDRLs were updated most recently in 2023, the data pool included scans as far back as 2012. The existing Australian NDRLs were divided into two age categories, 0–4 years and 5–14 years, whereas the local values found in this study have four age/weight categories (Table 3). The division of NDRLs into two age cohorts versus four likely allowed for a greater sample size in each cohort, though each represents a broader population than suggested by the European Commission.^6^ Our utilization of multiple narrow cohorts allows the resulting LDRL to represent the patient population more closely, though it resulted in fewer data points per cohort.

**Table 3.** Comparison of the local DRLs from this study with published data.

Country	Exam	Head				Chest				Abdomen/Pelvis			
	Age	<1	1–5	6–10	11–15	<1	1–5	6–10	11–15	<1	1–5	6–10	11–15
Australian LDRLs (this study)	CTDI_vol_	9.6	12.1	15.8	22.7	0.6	0.6	0.9	1.3	0.7	1.0	1.7	4.6
	DLP	156	206	282	399	10	16	26	43	16	39	71	230
Canadian LDRLs (this study)	CTDI_vol_	30.9	31.4	34.9	41.8	1.7	1.8	2.7	3.0	1.9	2.4	2.8	3.6
	DLP	539	611	718	859	30	45	76	102	52	76	107	169
Norwegian LDRLs (this study)	CTDI_vol_	30.0	36.8	40.9	41.3	1.1	1.6	2.1	2.9	1.1	1.5	3.7	8.1
	DLP	481	658	791	810	19	35	55	98	30	55	119	373
Australia NDRLs^13^	Age	0–4	–	5–14	–	0–4	–	5–14	–	0–4	–	5–14	–
	CTDI_vol_	30	–	35	–	2	–	5	–	7	–	10	–
	DLP	470		600		60		110		170		390	
Canada NDRLs^14^	Age	0–3	3–7	7–13	–	0–3	3–7	7–13	–	0–3	3–7	7–13	–
	CTDI_vol_	37	49	57	–	2.8	3.8	4.8	–	3.8	4.9	6.1	–
	DLP	578	843	888		62	87	136		120	185	263	
Nordic DRLs^15^	Age	0–3m	3m–<1	1–<6	>6	1m–<4	4–<10	10–<14	14–<18	–	4–<10	10–<14	14–<18
	CTDI_vol_	28	27	31	39	1.2	1.6	2.4	3.0	–	2.6	3.4	5.0
	DLP	403	398	516	679	25	41	64	103		92	150	247

^a^Different age groups were chosen for head examination.

The local DRLs suggested for the Canadian hospital were also below previously established Canadian NDRL values.^14^ The existing Canadian NDRLs include groups of patients aged 0–3 years, 3–7 years, and 7–13 years (Table 3), representing a different average patient age than reported in our study. This makes the direct comparison of established NDRLs to our suggested LDRLs challenging. It is important to note that existing Canadian pediatric NDRLs were published in 2016, based on the data collected in 2013, meaning they may not represent current technology, as updated CT scanners are more equipped for radiation dose reduction. While this is true, the Canadian data in this study came from a scanner installed in 2014; therefore, we can assume there are further opportunities for lower DRLs when considering the use of more modern equipment.

Pediatric DRLs established in the Nordic region in 2022 combined pediatric CT dose data from Denmark, Iceland, Norway, and Sweden.^15^ These are the only established pediatric CT DRLs incorporating Norwegian data. Nordic head CT DRLs were divided into 0–3 months, 3 months–<1 year, 1–<6, and >6 years patient age groups. For chest and abdomen/pelvis examinations, the Nordic values were categorized by weight and did not incorporate patient age.^15^ Using the approximate equivalent weight and age conversion chart provided by the European Commission,^6^ local DRLs from the Norwegian hospital for both chest and abdomen-pelvis CT scans follow closely with national values, demonstrating appropriate adherence to these Nordic DRLs, except for 11–15-year-old abdomen-pelvis examinations.^15^ In contrast, all head CTDI_vol_ and DLP values represented here exceeded that of Nordic DRLs, as shown in Table 3. This suggests the potential for optimization of pediatric head CT. Interestingly, while the Nordic DRL study was released in 2022, data collection took place between 2018 and 2019, meaning the Norwegian scanner sampled in our study represents the most modern equipment.

Our study showed that abdomen-pelvis reference levels from the Canadian hospital for patients aged 11–15 years were lower than those collected from Australian and Norwegian facilities (Figure 3). A possible explanation for this is differences in patient size. The median weight/thickness of patients aged 11–15 years receiving an abdomen-pelvis CT in Canada was 41.0 kg/16.8 cm; while in Australia and Norway, it was 50.0 kg/21.4 cm and 44.7 kg/19.5 cm, respectively (Table 2). The lower weight of Canadian patients in the 11–15-year-age cohort likely contributed to the low LDRL. The differences observed in this age group could also be explained by variability in protocol, such as the age or weight at which patients begin to be scanned under adult parameters.

In contrast, the CTDI_vol_ and DLP proposed as local DRLs at the Canadian hospital for pediatric chest CT exceeded that of Norwegian and Australian local reference values in all age cohorts, including the 11–15-year-old group, despite demonstrating similar differences in weight as in the abdomen-pelvis cohorts. The median weight of patients aged 11-15 years receiving a chest CT in Canada was 39.3 kg; while in Australia and Norway, it was 45.9 kg and 47.0 kg, respectively (Table 2). These noted differences in DRLs indicate the potential for optimization but must consider many other influences on examination dose such as operator use, operator training, scanner age, radiologist preference, diagnostic needs of the study, and image quality.

A patient’s size influences the dose modulation and radiation dose used during scanning; therefore, incorporating size into established DRL figures can ensure they accurately represent their assigned population. While CTDI_vol_ measures the scanner radiation output, SSDE accounts for patient size and better indicates actual patient dose.^17^ Our study demonstrated that SSDE values were higher than CTDI_vol_ in all age groups and considered examinations. Other studies have reported similar results for pediatric and small patients.^18–20^ Therefore, the displayed CT dose indices from pediatric scans significantly underestimate the patient’s radiation dose. Pediatric protocols are often based on patient weight, and a demonstrated strong positive correlation between SSDE and patient weight indicates that it might be a more accurate dosimetric parameter for protocol optimization. Moreover, SSDE can be automatically and retrospectively recorded compared to patient weight. In future studies of pediatric CT DRLs, we suggest that SSDE be incorporated as an indicator of radiation use.

While this study provides valuable insights into the local practices in three different countries, it is important to acknowledge the limitations regarding the representativeness of the involved hospitals. The selection of hospitals was based on existing academic relationships, which, while facilitating data collection and analysis, may not capture the full spectrum of practices and technological variations present in the country. Additionally, within each country, the data were collected from a limited number of hospitals, which may not fully represent national practices. A limited number of pediatric CT examinations performed within the prescribed time parameters in each age category resulted in a relatively low sample size in some cohorts, notably for the abdomen-pelvis CTs in the <1-year group. No image quality assessment was performed in this study; however, it was assumed that all examinations were of diagnostic quality because the data were collected retrospectively from examinations reported by radiologists.

Another noteworthy limitation of this study was the number of scanners included in the survey. This limited our ability to draw conclusions based on variables such as scanner type and protocol. Multiple independent researchers manually collected and recorded data, leading to potential human error or bias.

In conclusion, our study proposed LDRLs for pediatric CT examinations of the head, chest, and abdomen/pelvis. The resulting values reflect differences in practices, such as using CT only for ancillary imaging at the Australian facility. Our findings suggested that there are opportunities for optimization in pediatric CT imaging, particularly chest CT in Canada and head CT in Norway. Promoting automated and standardized data collection may minimize human error in future DRL studies. Furthermore, integrating SSDE as a standard metric for DRLs can help achieve a more accurate dose descriptor for pediatric patients, thus improving radiation safety and optimizing imaging practices in pediatric CT.
